# Supplementary material for: Transcriptome Analysis Reveals Vimentin-Induced Disruption of Cell–Cell Associations Augments Breast Cancer Cell Migration
Source: Cells. 2022 Dec 13;11(24):4035. doi: 10.3390/cells11244035 (PMC9776984; doi:10.3390/cells11244035)
Supplement: Supplementary file 1 [file cells-11-04035-s001.zip › DEGs and enrichment analysis.zip!/DEGs and enrichment analysis/WT_VIMvsVector KEGG/ALL/WT_VIMvsVector_ALL.pathway_map.zip!/WT_VIMvsVector_ALL.pathway_map/src/hsa00830.html]

hsa00830


- hsa:8854

- Down regulated genes

ENSG00000128918(-2.45255771806971)

- hsa:56603

- Down regulated genes

ENSG00000003137(-2.14650186746975)
- hsa:1592

- Down regulated genes

ENSG00000095596(-2.14232529713165)

- hsa:56603

- Down regulated genes

ENSG00000003137(-2.14650186746975)
- hsa:1592

- Down regulated genes

ENSG00000095596(-2.14232529713165)

- hsa:1592

- Down regulated genes

ENSG00000095596(-2.14232529713165)

- hsa:9249

- Down regulated genes

ENSG00000162496(-2.52856145206977)

- hsa:56603

- Down regulated genes

ENSG00000003137(-2.14650186746975)
- hsa:1592

- Down regulated genes

ENSG00000095596(-2.14232529713165)

- hsa:1555

- Down regulated genes

ENSG00000256612(-3.39526895468382)

- hsa:1555

- Down regulated genes

ENSG00000256612(-3.39526895468382)

- hsa:1555

- Down regulated genes

ENSG00000256612(-3.39526895468382)

- hsa:1555

- Down regulated genes

ENSG00000256612(-3.39526895468382)

- hsa:8854

- Down regulated genes

ENSG00000128918(-2.45255771806971)

Close
